# SARS-CoV-2 Exacerbates COVID-19 Pathology Through Activation of the Complement and Kinin Systems

**DOI:** 10.3389/fimmu.2021.767347

**Published:** 2021-11-05

**Authors:** Anne G. Savitt, Samantha Manimala, Tiara White, Marina Fandaros, Wei Yin, Huiquan Duan, Xin Xu, Brian V. Geisbrecht, David A. Rubenstein, Allen P. Kaplan, Ellinor I. Peerschke, Berhane Ghebrehiwet

**Affiliations:** ^1^ Department of Microbiology & Immunology, Renaissance School of Medicine of Stony Brook University, Stony Brook, NY, United States; ^2^ Department of Medicine, Renaissance School of Medicine of Stony Brook University, Stony Brook, NY, United States; ^3^ Department of Biomedical Engineering, Stony Brook University, Stony Brook, NY, United States; ^4^ Department of Biochemistry and Molecular Biophysics, Kansas State University, Manhattan, KS, United States; ^5^ Pulmonary and Critical Care Division, The Medical University of South Carolina, Charleston, SC, United States; ^6^ The Department of Laboratory Medicine, Memorial Sloan Kettering Cancer Center, New York, NY, United States

**Keywords:** Sars-CoV-2, complement, bradykinin, COVID-19, kinin-kallikrein system, post COVID “long-haulers”

## Abstract

Infection with SARS-CoV-2 triggers the simultaneous activation of innate inflammatory pathways including the complement system and the kallikrein-kinin system (KKS) generating in the process potent vasoactive peptides that contribute to severe acute respiratory syndrome (SARS) and multi-organ failure. The genome of SARS-CoV-2 encodes four major structural proteins – the spike (S) protein, nucleocapsid (N) protein, membrane (M) protein, and the envelope (E) protein. However, the role of these proteins in either binding to or activation of the complement system and/or the KKS is still incompletely understood. In these studies, we used: solid phase ELISA, hemolytic assay and surface plasmon resonance (SPR) techniques to examine if recombinant proteins corresponding to S1, N, M and E: (a) bind to C1q, gC1qR, FXII and high molecular weight kininogen (HK), and (b) activate complement and/or the KKS. Our data show that the viral proteins: (a) bind C1q and activate the classical pathway of complement, (b) bind FXII and HK, and activate the KKS in normal human plasma to generate bradykinin and (c) bind to gC1qR, the receptor for the globular heads of C1q (gC1q) which in turn could serve as a platform for the activation of both the complement system and KKS. Collectively, our data indicate that the SARS-CoV-2 viral particle can independently activate major innate inflammatory pathways for maximal damage and efficiency. Therefore, if efficient therapeutic modalities for the treatment of COVID-19 are to be designed, a strategy that includes blockade of the four major structural proteins may provide the best option.

## Introduction

Coronaviruses (CoVs) are a group of related viruses which cause mild to severe diseases in both humans and animals. However, 3 of the last 7 pathogenic coronaviruses reported have caused much more severe and often fatal respiratory infections in humans and have been responsible for deadly pneumonia outbreaks in the 21st century (1–9). Coronaviruses cause a lethal disease called severe acute respiratory syndrome (SARS), in which the subsequent edema in the lungs prevents oxygen uptake, resulting in deadly hypoxia (4, 5). Since the first major outbreak of SARS in 2002, there have been two major coronavirus pandemics: MERS-CoV (Middle Eastern Respiratory Syndrome Coronavirus) in 2012, which affected 27 countries in the Middle East, Africa and South Asia, and the present COVID-19 pandemic, which is caused by SARS-CoV-2 (1–9). Therefore, as the virus adjusts and adapts to its environment, it will certainly mutate through either immunologic shift or immunologic drift, releasing respectively new strains or variants that cause novel pandemics in the future (1–9). In fact, the new variants of SARS-CoV-2 that appeared very recently in the UK and S. Africa and now also showing up in the US and other parts of the world are almost certainly the beginning of what is to come. Therefore, complete understanding of the molecular structures and the mutations that trigger and/or exacerbate the diseases caused by SARS-CoV-2 may help us identify novel pharmacological targets for the development of therapies that challenge present as well as future pandemics.

The genome of the SARS-CoV-2 encodes four major structural proteins: the spike (S) protein, nucleocapsid (N) protein, membrane (M) protein, and the envelope (E) protein, all of which are required to produce a structurally complete viral particle (1, 2). Among these structural proteins however, the S protein takes center stage in SARS-CoV-2 infection as it is singularly responsible for viral attachment, fusion, and entry into target cells. Infection with SARS-CoV-2 is initiated when the spike (S) protein interacts with its cognate host cell surface receptor (5, 10). SARS-CoV-2 infects human lung alveolar type II epithelial cells by attaching *via* its S protein to angiotensin converting enzyme 2 (ACE-2) expressed at the cell surface (9). Viral entry is facilitated when the type II transmembrane serine protease (TMPRSS2) cleaves the viral S protein into S1 and S2, with the latter causing membrane fusion (7–9). This is followed by simultaneous activation of powerful, cross-reactive inflammatory pathways in plasma resulting in rapid production of vasoactive peptides, which in turn recruit leukocytes which secrete inflammatory cytokines that contribute to the vascular leakage and edema culminating in severe acute respiratory syndrome. Foremost among these cross-reactive innate pathways are the complement system, the coagulation system, and the kallikrein-kinin system (KKS), each of which is able to generate activation byproducts that collectively contribute to the ‘cytokine storm’, multi-organ inflammation, bilateral pneumonia, and progression to the acute respiratory distress syndrome (ARDS) requiring ventilatory support (5, 9–13). The question is: which of the SARS-CoV-2-associated molecular structures are responsible for the activation of the innate immune pathways?

The present studies were undertaken to examine in detail if any of the highly conserved SARS-CoV-2 proteins–which are encoded by all the coronaviruses–can activate the complement and/or the kinin system. Furthermore, since gC1qR (14, 15), the receptor for globular heads of C1q as well as for high molecular weight kininogen (HK), is often overexpressed and released by infected cells, we hypothesized that, if any of the SARS-CoV-2 structural proteins bind gC1qR, then a virus decorated with gC1qR, or released viral proteins bound to gC1qR, may provide a suitable complex for the assembly and activation of the complement system, the KKS and–directly or indirectly–the coagulation system (9, 11–13). Therefore, we also examined if any of the SARS-CoV-2 structural proteins directly interact with gC1qR in a manner that activates the complement system and/or the KKS.

## Materials and Methods

### Chemicals and General Reagents

Recombinant proteins corresponding to the structural proteins of SARS-CoV-2 (S1, N and a fusion protein of M-E), were purchased from ViroGen Corporation (Brighton, MA), and the M and E proteins were purchased from MyBioSource, Inc. (San Diego, CA), as was biotinylated anti complement C4d fragment. Bradykinin (BK) ELISA kit was purchased from Enzo Life Sciences Inc. (East Farmingdale, NY), and normal human plasma from random donors was purchased from Oklahoma Blood Institute (Oklahoma City, OK).

### Expression of Recombinant gC1qR

The strategies for expression and purification of mature gC1qR and deletion mutants have been described previously (14–19).

### Solid-Phase Microplate Binding Assay

The interaction of viral proteins with gC1qR was assessed by a standard ELISA. Briefly, microtiter plate wells were coated in duplicates (60 min, at 37°C or overnight, 4°C) with 100 µl of either, concentrations of viral proteins (S1, N, M, E or a fusion M-E) ranging from 2-10 µg/ml, or heat inactivated BSA, in carbonate buffer, pH 9.6 (15 mM Na_2_CO_3_ and 35 mM NaHCO_3_). The unbound protein was removed from each well; the wells washed 2x with TBST (20 mM Tris-HCl pH 7.5, 150 mM NaCl, and 0.05% Tween-20), and the unreacted sites blocked by incubation (30 min, room temp) with 300 µl of 1% heat inactivated BSA (1hr, 37°C). After washing (2x with TBST), the microtiter plate bound proteins were incubated (1hr, room temp.) with concentrations of biotinylated gC1qR, ranging from 0 to 5 µg/ml. This was followed by sequential reaction (1 hr each, room temp) with alkaline-phosphatase conjugated streptavidin and pNPP, and the color developed at the end of the incubation read spectrophotometrically at 405nm.

Similarly, the binding of FXII or HK to microtiter plate coated S1, N, M-E was performed using the same strategy and the bound FXII or HK was detected using specific antibodies. In all cases, each experiment was repeated at least three times in duplicates (n=3).

### Surface Plasmon Resonance

Biosensor surfaces were prepared by immobilizing either wild-type gC1qR (i.e., gC1qR-WT) or a gC1qR deletion mutant that removed the flexible, negatively charged loops (i.e., gC1qR-ΔΔ). Then, a two-fold dilution series of recombinant forms of various SARS-CoV-2 structural proteins was injected over each surface. The reference-corrected sensorgrams were then fit to kinetic models to obtain the apparent equilibrium dissociation constant for each interaction pair. Of note, the gC1qR-ΔΔ used in these studies (not published) was generated–as an alternate to gC1qR-WT for crystallographic experiments–by removing the highly charged D and E rich loops. Interestingly, despite the missing charged loops, we found that the gC1qR-ΔΔ not only crystallizes readily, but also forms a trimer both in solution and crystal, demonstrating that the protein is indeed intact. More importantly, HK binds to both gC1qR and gC1qR-ΔΔ equally well (unpublished data).

### Hemolytic Assays for Complement Activation

To assess whether the viral antigens that bound to C1q also activate complement in serum, we used a standard hemolytic assay for complement activity using AggIgG as a positive control. To make AggIgG, 10 mg/ml of IgG was incubated at 62°C for 20 min and then the large precipitates formed were removed by centrifugation at 1000xg for 30 min. Each AggIgG was then tested for complement activation before use (20). Briefly, 10 µl of normal human serum (NHS, Complement Technology, Inc., Tyler, TX) in 100 µl GVB^++^ (gelatin containing veronal buffer) was first incubated (1hr, 37°C) with or without various concentrations of recombinant viral proteins S1, N, M, E, or the fusion of M-E. As a positive control for complement activation, NHS was incubated with 10 µl of aggregated IgG (AggIgG) in 100 µl of GVB^++^. After incubation, 50 µl of sensitized sheep erythrocytes (EAs) (2x10^8^/ml sheep red blood cells sensitized with anti-sheep IgG) were added to each tube, the volume brought up to 500 µl with GVB^++^ and further incubated (1hr, 37°C). The tubes were then centrifuged and the degree of hemolysis in the supernatant in each tube determined spectrophotometrically at 412 nm. The total, releasable hemoglobin (100%), was achieved by lysis of 50 µl EAs with 450 µl of H_2_O, against which the hemoglobin released in each experimental tube was compared.

To assess the deposition of complement C1q and C4d fragment on viral proteins under the conditions of the hemolytic assay, viral proteins were coated in wells of microtiter plates (10 µg/mL) and blocked with 1% heat inactivated BSA as described under Solid Phase Microtiter Plate Assay, above. Wells were then incubated with 50 µL of a 1:10 dilution of NHS in GVB^++^ and incubated at 37°C for 2 hrs. After washing, the wells were then probed with either biotinylated anti C4d or goat anti C1q in GVB^++^, followed by streptavidin AP or rabbit anti goat AP. Following washing with GVB^++^, wells were incubated with pNPP and color development monitored at 405 nm.

### Activation of the KKS by Viral Proteins

To test if the binding of the viral proteins resulted in KKS activation, we used a commercial BK ELISA kit (Enzo Life Sciences Inc., East Farmingdale, NY) and followed the manufacturer’s recommendations. Briefly, 1 ml of normal human plasma (from random donors, purchased from Oklahoma Blood Institute, Oklahoma City, OK) diluted 1:16 in assay buffer (with 50 µM ZnCl_2_) was mixed with various concentrations (1, 2, 5 or 10 µg/mL) of S, M, and N proteins and incubated at 37°C for 1 hour. The plasma samples were then diluted and added to rabbit-IgG antibody-coated wells, along with biotin-conjugated bradykinin and a rabbit polyclonal antibody to bradykinin. Through a competitive binding process, biotin-conjugated bradykinin was captured on the bottom of the wells, which was detected using horseradish peroxidase-conjugated streptavidin. Color development was read at 405nm in a Spectramax i3x microplate reader (Molecular Devices).

### Statistical Analysis

Student t-tests were performed using statistical software (Excel; Microsoft, Redmond, WA, USA). A value of p=0.05 was a significant difference. (n - represents number of separate experiments performed in duplicates).

## Results

### Activation of the Classical Pathway of Complement by SARS-CoV-2 Proteins

**Figure 1:**
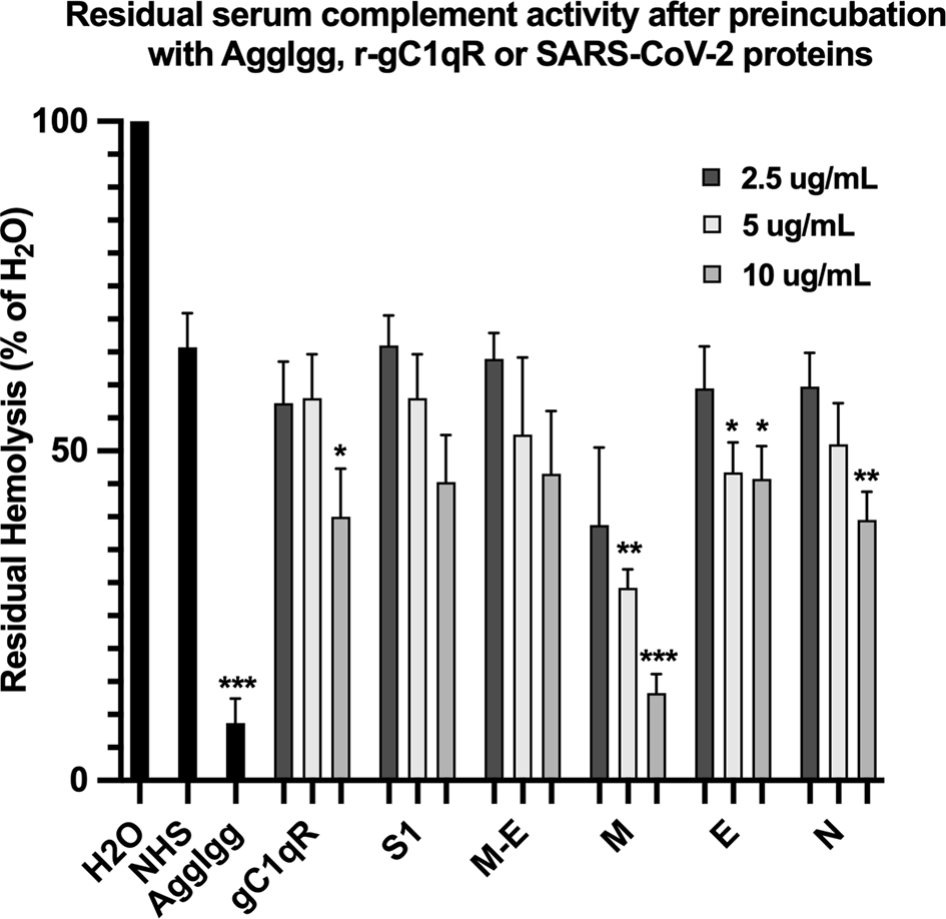
Hemolytic assay to assess the effect of SARS-CoV-2 recombinant proteins on complement activity. Recombinant spike (S1), membrane (M), envelope (E) and nucleocapsid (N) proteins were pre-incubated with NHS in GVB^++^ (1:10 dilution) for 1 hr at 37°C. Then EAs were added and incubated continued for an additional hour. Cells were removed by centrifugation and the absorbance of the hemoglobin released into the supernatants was read at 412 nm. Positive control for complement activity is NHS incubated with EAs, and control for complement consumption is NHS pre-incubated with AggIgG and then with EAs. Data are representative of three independent experiments (n=3). Results are expressed as percent hemolysis relative to 100% (H_2_0). Statistical analysis unpaired T test with Welch’s correction was performed in Graphpad Prism. Error bars represent 1SD. *p ≤ 0.05; **p ≤ 0.01, ***p ≤ 0.001.

**Figure 2:**
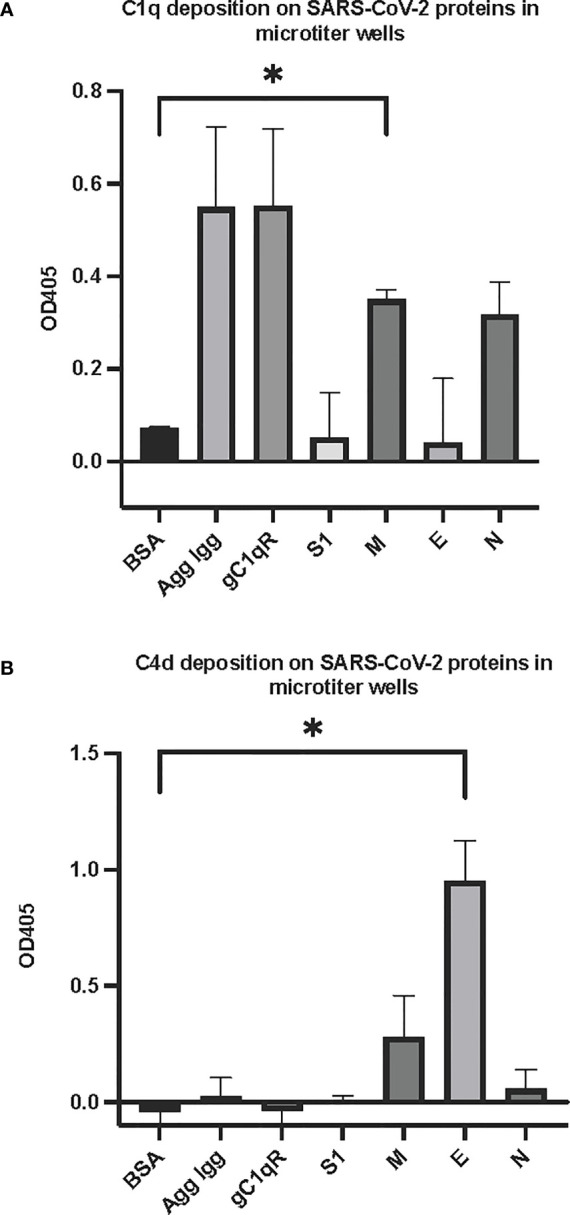
Deposition of complement proteins **(A)** C1q and **(B)** C4d fragment on SARS-CoV-2 proteins immobilized on microtiter plates. Microtiter wells were coated with test proteins at 10 μg/mL. Following blocking with inactivated BSA, the wells were washed with GVB^++^ followed by application of NHS (1:10 dilution in GVB^++^) and incubation at 37°C for 2 hr. After washing, goat anti human C1q antibody **(**panel **A)** or biotinylated anti C4d polyclonal antibody **(B)** was applied for 1 hr, then wells were incubated with streptavidin-AP **(A)** or rabbit anti goat AP **(B)** and proteins detected with the AP substrate pNPP. Statistical analysis on all samples compared to BSA negative control, unpaired T test with Welch’s correction was performed in Graphpad Prism. Error bars represent 1SD. *p ≤ 0.05.

The first question we asked was: do the viral proteins trigger activation of the classical pathway of complement? As shown in **Figure 1**, preincubation of each of the viral structural proteins with normal human serum results in a dose-dependent diminished complement activity. The diminished hemolytic activity in turn, is due to consumption of complement as a result of preincubation of the viral proteins with NHS. However, since preincubation of the viral proteins with NHS could also result in inhibition of complement due to blockade of C1q or other proteins critical in complement activation, we performed two additional experiments to confirm a *bona fide* activation of the cascade. First, we showed that incubation of the viral proteins with C1q-depleted serum did not result in complement activation (not shown), suggesting that the classical pathway is activated by the SARS-CoV-2 structural proteins. Second, we confirmed that incubation of NHS to microtiter bound viral proteins results in C1q deposition (**Figure 2A**), followed by the presence of degradation fragments such as C4d (**Figure 2B**), which confirms the activation of the classical pathway of complement. The fact that the viral proteins bind to C1q in a specific and dose-dependent manner, followed by the experiment in which C1q-depleted serum did not result in complement activation suggests that it is the classical pathway and not the MBL pathway that is activated under these conditions. This distinction is important since recent studies have also shown that the MBL pathway, which is similar to the classical pathway in its mode of activation, can be activated by SARS-CoV-2 proteins (21).

### SARS-CoV-2 Proteins Bind HK, and FXII and Generate BK

**Figure 3:**
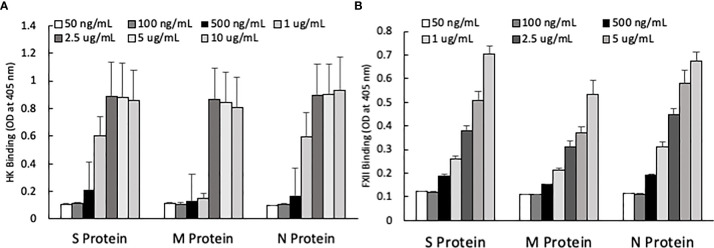
**(A)** High molecular weight kininogen (HK) binding to immobilized S, M and N protein at different concentrations. **(B)** Factor XII (FXII) binding to immobilized S, M and N protein at different concentrations. Data is presented as mean + standard error (n=3-8 for HK binding, and n=4 for FXII binding). ANOVA indicates that the viral protein concentration has a significant effect on HK binding (*P*=0.0033 for S protein, *P*=0.0045 for M protein, and *P*=0.0163 for N protein) and FXII binding *(P*=0.0001 for all three proteins).

**Figure 4:**
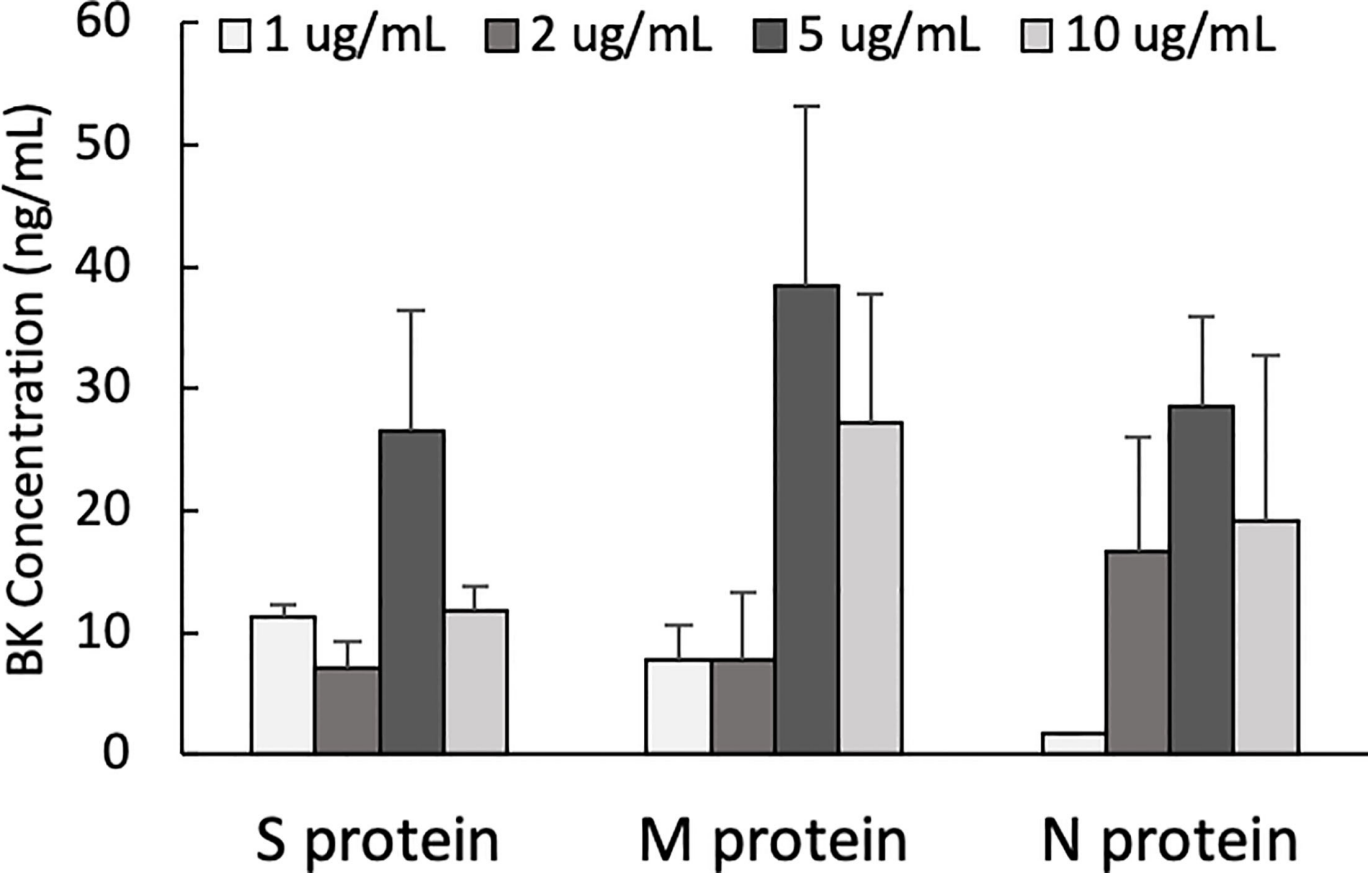
Bradykinin assay. Bradykinin concentration (ng/mL) in normal human plasma incubated with S, M and N protein at 37°C for 1 hour. Data is presented as mean + 1SD (n=2-3.).

One of the mechanisms by which edema formation can occur in COVID-19 patients is through the activation of the KKS and the subsequent formation of bradykinin (BK). However, for this to occur, the viral proteins would have to bind to the key components in KKS, which are HK and FXII. Therefore, we tested whether any of the viral proteins could bind to these proteins. As shown in **Figure 3**, both HK (A) and FXII (B) dose-dependently (50 ng/ml to 10 μg/ml) bind to immobilized proteins of SARS-CoV-2. Binding increases relative to the concentration of the added protein with a p ≤ 0.0045 for HK and p ≤ 0.0001 for FXII. More importantly, incubation of the viral proteins with normal human plasma in the presence of 50 µM ZnCl_2_ results in the generation of BK (**Figure 4**), which in turn is dependent on the amount of viral protein added to the plasma. At the highest concentration of each viral protein added, there is a diminished BK generation. However, this may be because at high concentration, there is a rapid generation of BK, followed by rapid enzymatic degradation consistent with the half-life of BK in plasma, which is <1 min. Therefore, the degraded BK fragments may not be recognized by the antibody to BK, thereby giving the false impression that higher concentrations of viral proteins give rise to the formation of fewer molecules of BK.

### Binding of SARS-CoV-2 Structural Proteins to gC1qR

**Figure 5:**
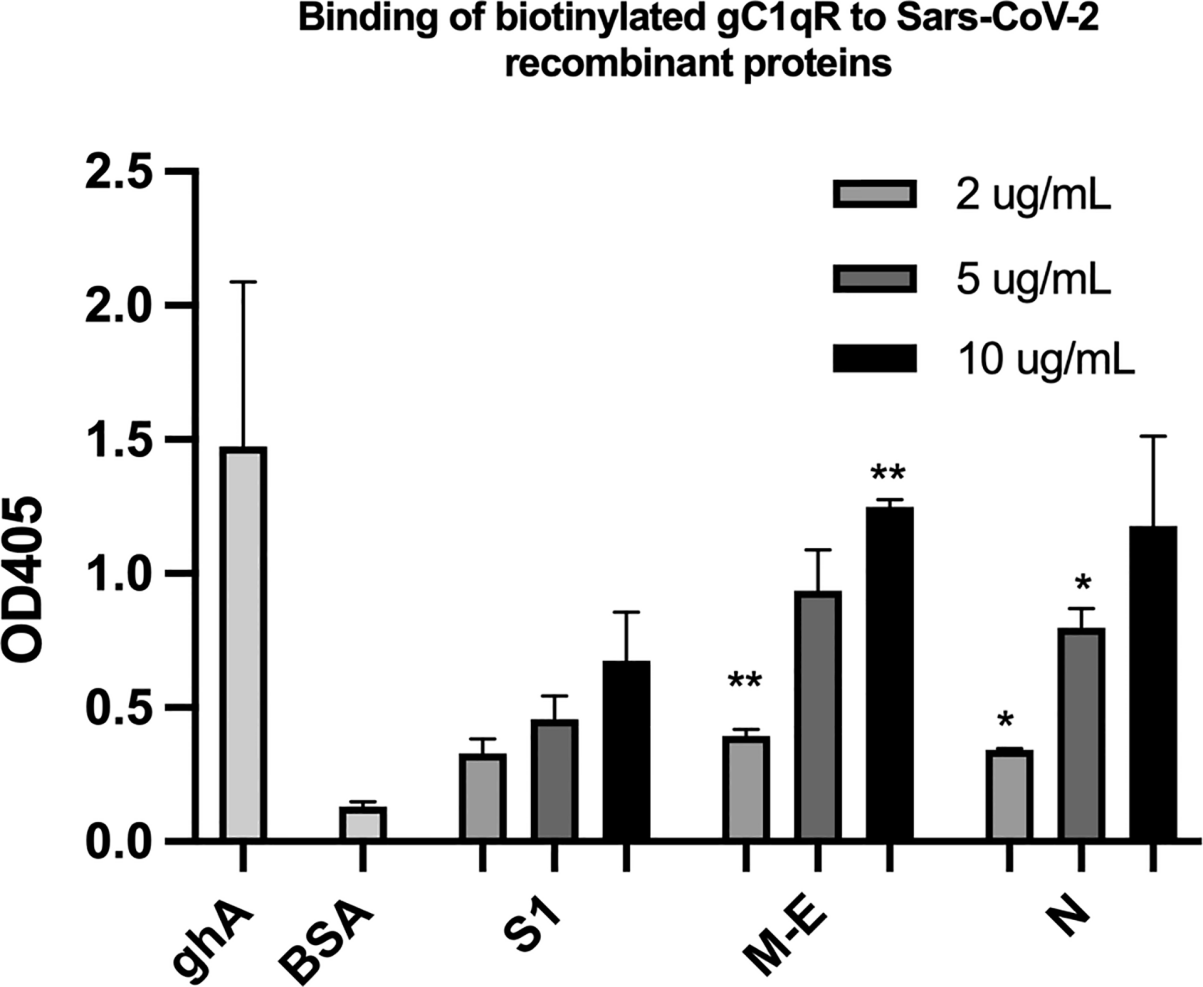
Binding of biotinylated gC1qR to SARS-CoV-2 structural proteins immobilized on microtiter plates. Microtiter plate wells were coated with the indicated amount of test protein, and after blocking unreacted sites with BSA, biotinylated gC1qR at a concentration of 1 ug/ml was added and the bound gC1qR detected by incubation with AP-streptavidin. Positive control for binding is ghA (the A chain of the globular head of C1q) and negative control for non-specific binding is BSA. Statistical analysis comparing , each data point with the BSA negative control is the unpaired T test with Welch’s correction was performed in Graphpad Prism (n=3; and *p ≤ 0.05, **p ≤ 0.01).

Since soluble or cell surface gC1qR can activate the classical pathway of complement and the kinin-kallikrein system (KKS)–both of which play a significant role in the pathogenesis of COVID-19–we postulated that if any of the SARS-CoV-2 structural proteins bind gC1qR, then the gC1qR-decorated viral particles could potentially provide a platform for the simultaneous activation of both the complement and the KKS pathways. To test this hypothesis, we first used solid phase ELISA to test if the viral proteins bind to gC1qR. As shown in **Figure 5**, all of the SARS-CoV-2 structural proteins bind to gC1qR in a dose-dependent manner. Since gC1qR is the receptor for the globular head of the A chain of C1q, recombinant ghA was used as a positive control for gC1qR. Furthermore, the binding of the viral proteins is enhanced in the presence of 50 µM Zn^+^ (not shown) suggesting that the viral protein-gC1qR interaction may be zinc ion-dependent in a manner similar to the interaction between FXII and HK to gC1qR (16–19).

**Figure 6:**
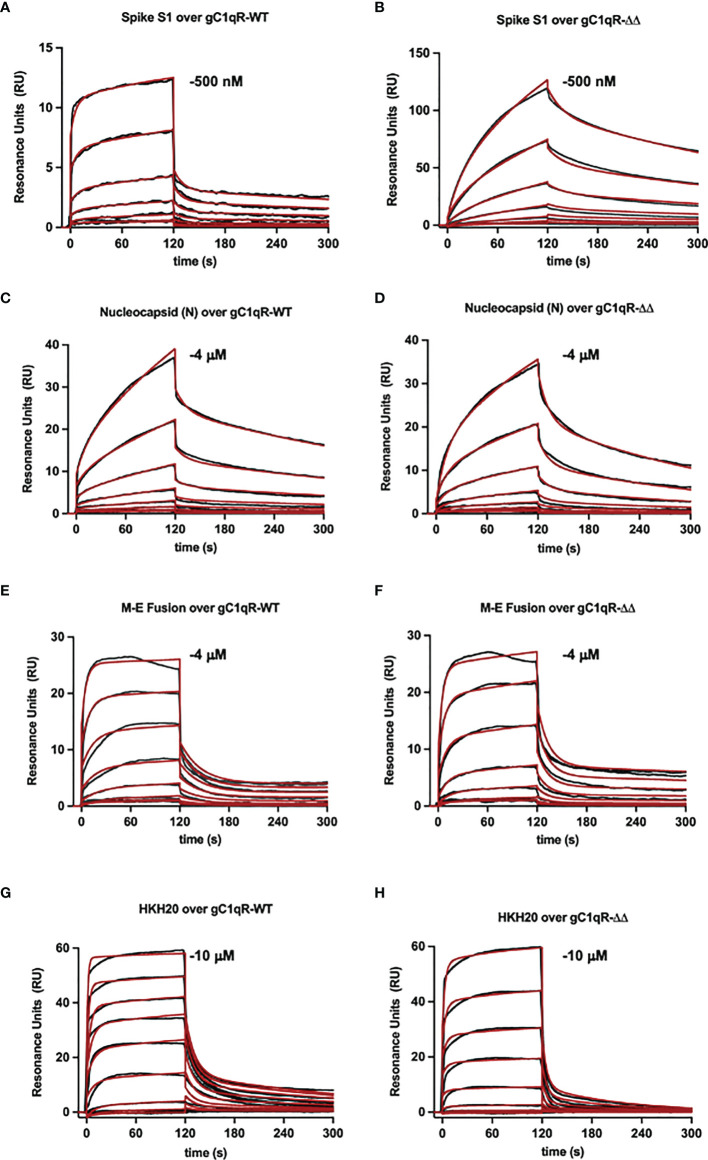
Surface Plasmon Resonance. Surfaces were prepared by immobilizing either wild-type gC1qR (i.e. gC1qR-WT) or a gC1qR deletion mutant that removed the flexible, negatively charged loops (i.e. gC1qR-ΔΔ). A two-fold dilution series of recombinant forms of various SARS-CoV-2 structural proteins was injected over each surface, where the highest concentration of each protein used is inset. The reference-corrected sensorgrams (black traces) were fit to kinetic models (red traces) to obtain the apparent equilibrium dissociation constant for each interaction pair. Representative sensorgrams from 3 independent determinations are shown for **(A, B)** Spike-S1 protein over gC1qR-WT (K_D_=91 nM) or gC1qR-ΔΔ (K_D_=370 nM), **(C, D)** Nucleocapsid protein over gC1qR-WT (K_D_=6 µM) or gC1qR-ΔΔ (K_D_=12 µM), **(E, F)**, Membrane-Envelope Fusion protein over gC1qR-WT (K_D_=410 nM) or gC1qR-ΔΔ (K_D_=360 nM), and **(G, H)** a synthetic peptide corresponding to the gC1qR-binding region from HK as a positive control (K_D_=2 mM and 10 mM, respectively).

To examine the interaction between gC1qR and the viral proteins through an independent approach, we also used surface plasmon resonance binding assay. In these experiments, purified viral proteins were injected over surfaces of either wild-type gC1qR or a deletion mutant that removed the two disordered negatively charged loops found in the gC1qR sequence (i.e., gC1qR-ΔΔ). Representative series of sensorgrams from at least three independent determinations are shown in **Figure 6**. Apparent binding affinities for each interaction are as follows: (A, B) Spike-S1 protein over gC1qR-WT (K_D_=91 nM) or gC1qR-ΔΔ (K_D_=370 nM), (C, D) Nucleocapsid protein over gC1qR-WT (K_D_=6 µM) or gC1qR-ΔΔ (K_D_=12 µM), (E, F), Membrane-Envelope Fusion protein over gC1qR-WT (K_D_=410 nM) or gC1qR-ΔΔ (K_D_=360 nM), and (G, H) a synthetic peptide corresponding to the gC1qR-binding region from HK (19, 20) as a positive control (over gC1qR-WT, K_D_=2 mM or over gC1qR-ΔΔ, 10 mM). Together, these observations revealed that two different forms of gC1qR bind the various SARS-CoV-2 structural proteins we tested with affinities in the ~0.1-10 µM range. Furthermore, since SPR analyses were conducted using purified components, our results strongly suggest that these interactions occur directly and can take place in the absence of contributions from other proteins.

## Discussion

**Figure 7:**
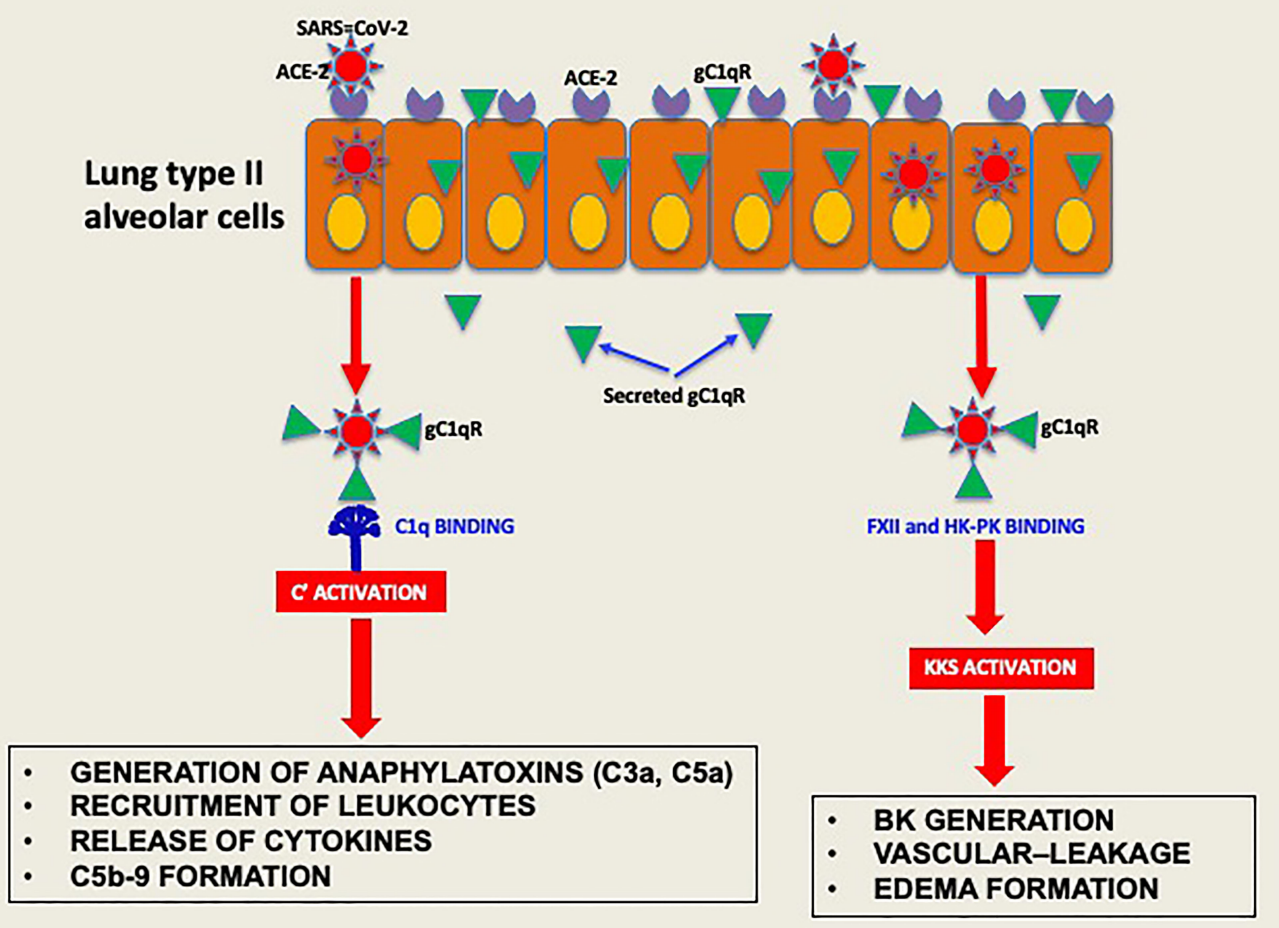
Hypothetical role of gC1qR as an activator of complement and KKS. After the initial infection, multiple copies of the virus leave the primary cell and upon release bind to secreted gC1qR, a ‘self’ protein, to evade the immune system. The virus decorated with gC1qR in turn serves as an efficient platform for the activation of both the complement system and the kinin system to generate vasoactive peptides such as C3a, C5a and BK.

Experimental evidence available to date supports the concept that the pathogenesis induced by SARS-CoV-2 infection is largely due to the simultaneous activation of several cross-reactive immune pathways (2, 20–26) that generate in the process potent and multifunctional activation products that collectively contribute to severe inflammation, intravascular thrombosis, excessive edema, and eventually death (5, 8, 9, 11–13). One of the most consistent laboratory findings associated with the severity of COVID-19 is the elevated levels of D-dimer, which is produced when plasmin dissolves blood clots through a process called fibrinolysis (20, 21). However, in addition to its important role in fibrinolysis, plasmin has many other functions outside its conventional role including activation of FXII (27). For example, plasmin can activate proteins of the complement system and releases powerful bioactive fragments such as C3a and C5a (**Figure 7**), which in turn cause vascular permeability and recruitment of leukocytes that produce proinflammatory cytokines, thus contributing to the cytokine storm that is often associated with COVID-19 disease. In addition, the role of plasmin in activating FXII in the KKS, resulting in release of BK, has long been recognized (28, 29), and has recently been gaining more attention [reviewed in (27)].

Although advances have been made since the onset of the COVID-19 pandemic, the viral and host molecular networks that interact to trigger activation of the innate pathways (20, 21) and exacerbate the COVID-19 pathology are still poorly understood. As explained earlier, the major SARS-CoV-2 structural proteins are: the spike (S) protein, the nucleocapsid (N) protein (1, 2, 22–26, 30). the membrane (M) protein (2, 22–26, 30), and the envelope (E) protein (23, 26). Although all of these proteins are required to produce a structurally complete and highly pathogenic viral particle (1, 2), it is the S protein that is responsible for viral attachment to cells, fusion of viral and cellular membranes, and entry into cells, thus causing a full-blown SARS-CoV-2 infection (9, 10, 22). Not surprisingly therefore, this protein has been the focus of extensive studies as well as the major target for vaccine production.

Infection with SARS-CoV-2 is initiated when the S protein interacts with ACE-2 on the epithelial cell surface (9, 22). However, although the primary function of ACE-2 is to control the activity of ACE-1 in the renin-angiotensin system (RAS), another critical function of ACE-2 is the degradation of BK into non-functional peptides. Since BK generation is at the center of the edema formation in COVID-19 pathology, occupancy of ACE-2 by the viral S protein would inadvertently interfere with its ability to degrade BK thus leaving unregulated and active BK to circulate freely. Furthermore, activation of the kinin system also generates in the process multiple activation peptides including HKa, a cleavage product of HK, which binds gC1qR and promotes the release of cytokines such as TNFα, IL-1β, IL-6, and the chemokines IL-8 and MCP-1 from human mononuclear cells (31–37). Moreover, gC1qR secreted by infected cells has been shown to act as an autocrine signal to induce the expression of the high affinity receptor for HK, namely the bradykinin receptor 1 (B1R) (38). Together, these events would contribute to the exacerbation of the inflammatory processes associated with COVID-19.

Previous studies have shown that some of the SARS-CoV-2 associated proteins, and in particular the S protein, can activate the complement system (31–37) either *via* the MBL pathway (35) or the alternative pathway (37). In the present study, we show that all of the viral proteins also activate complement *via* the classical pathway, presumably by binding to the globular heads of C1q (gC1q) in a manner similar to IgG or gC1qR, which are also able to activate complement (38–41).

By virtue of its ability to bind and activate (29, 42, 43) two of the most potent inflammatory systems in plasma–the complement system and the kinin-kallikrein system (KKS)–we hypothesized that cell surface expressed or secreted gC1qR could also contribute to the rapid inflammatory response and the “cytokine storm” that is associated with COVID-19 pathology. The rationale for this postulate, in turn, is based on the fact that a diverse array of viruses such as HIV-1 (19), hepatitis C virus (44), bovine circovirus (45) Hantavirus (46), Epstein-Barr virus (47), and rubella virus (48) target gC1qR, inside and outside the cell for cellular entry and to enhance their own survival. For example, while infection with human papillomavirus (HPV) triggers gC1qR signaling and mitochondrial dysfunction and apoptosis (49), vesicular stomatitis virus induces gC1qR signaling to block retinoic acid-inducible gene I (RIG-I) activation thereby promoting its replication (50). Moreover, gC1qR expression and secretion is enhanced as a consequence of viral infection. Although it is not yet known whether SARS-CoV-2 engages gC1qR inside the cell, the ELISA and SPR binding data seem to suggest that it would.

**Figure 8:**
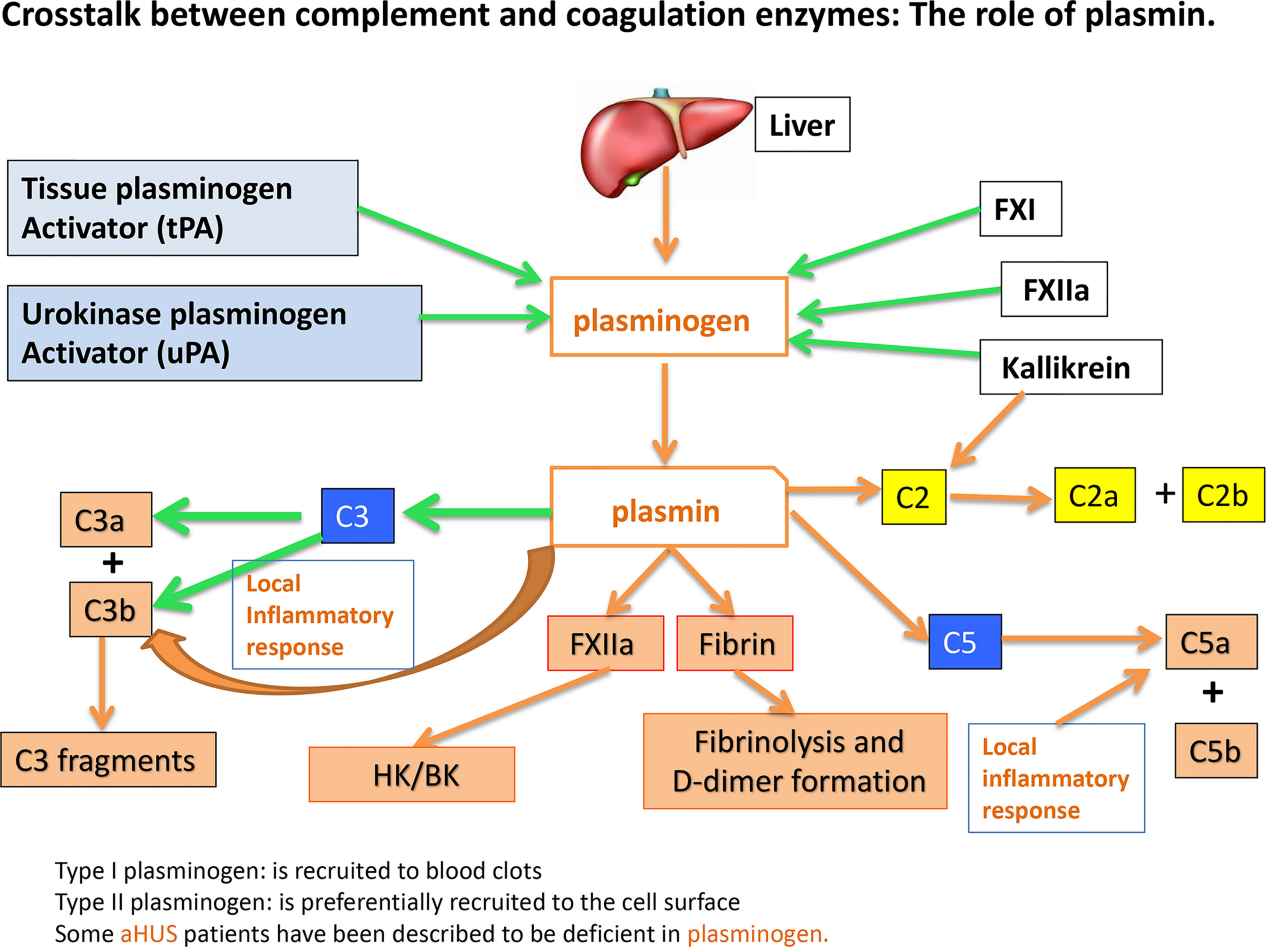
Crosstalk between complement and coagulation enzymes: The role of plasmin.

To survive and multiply, SARS-CoV-2, like many other viruses, has a built-in strategy that takes advantage of proteins of the host cell, to enter the cell. However, after dividing inside the initial host cell, new viruses are released and ready to invade other cells. But first, they must escape recognition by the immune system using self-molecules as a camouflage. By virtue of its abundance and affinity for pathogenic microorganisms, intracellular or secreted gC1qR may bind SARS-CoV-2 and thus prevent it from recognition by the immune system. In addition, the gC1qR-coated virus could serve as a platform for the assembly and activation of both the complement and the KKS pathway (39). Activation of the two systems (**Figure 8**) would then result not only in the generation of bradykinin, but also generation of activation fragments from the complement system such as C3a and C5a. In addition, the secreted gC1qR itself can induce the expression of B1R, the high affinity receptor for BK, thus providing the requisite receptor for vascular leakage, recruitment of leukocytes and secretion of cytokines. In addition to C1q, the plasma proteins that bind gC1qR are mostly blood coagulation proteins and include high molecular weight kininogen [HK], Factor XII [Hageman factor], fibrinogen, thrombin [FII], and multimeric vitronectin (38, 43). Although HK and factor XII compete for binding to gC1qR (51), recent crystallization of the complexes (52) show that the simultaneous binding of both to gC1qR, which is zinc dependent (18, 52) is possible. The involvement of the bradykinin forming cascade is shown by the findings that bronchoalveolar lavage studies of COVID-19 patients reveal marked upregulation of kallikreins, HK, and bradykinin receptors with downregulation of C1 inhibitor, ACE, ACE-2 (53) and improvement in oxygenation with a B-2 receptor antagonist (18, 53). Therefore, by binding to these proteins, gC1qR can play an additional role in COVID-19 pathology by modulating the crosstalk between the complement and coagulation pathways through fibrin formation, immune injury and/or inflammation The fact that all of the major structural proteins bind gC1qR efficiently therefore suggests that SARS-CoV-2 could potentially use gC1qR as an alternate receptor for cellular entry and/or intercellular communication, thus making gC1qR a pluripotent target exploited by SARS-CoV-2. More importantly, since gC1qR is also localized intracellularly, there is a potential for intracellular interaction between gC1qR and SARS-CoV-2 proteins (54, 55).

By virtue of its significance in cellular entry, the S protein has been the major focus of anti-SARS-CoV-2 vaccine production. However, the finding that all known viral structural proteins bind and activate innate pathways suggests that SARS-CoV-2 is probably one of the most efficient viruses capable of using its structural proteins for maximal damage. This in turn suggests that SARS-CoV-2 may hijack gC1qR as an alternate receptor for cellular entry, activation of innate pathways or intercellular communication and energy metabolism (54). More importantly, if the viral proteins are released and could survive in plasma or other tissues such as on platelet microparticles (56, 57), complement activation and BK generation could continue to occur even long after the virus has been cleared and severe symptoms associated with the initial infection have disappeared as is the case in the so called “long haulers”. Although they test negative for the SARS-CoV-2, approximately 10% of these patients develop a myriad of post-COVID-19 lingering symptoms that include incessant coughing, shortness of breath, body aches, brain fog, headaches, and joint pain. We speculate that fragments of the virus or the viral proteins may still linger in the blood possibly bound to self-molecules such as gC1qR, which would then continuously activate the complement system, and the KKS, thus releasing vasoactive and inflammatory molecules that contribute to the multi-system inflammation of “long-haulers”.

Our results not only reveal novel molecular correlates involved in the induction and/or enhancement of the “cytokine storm”, vascular permeability and edema that are the hallmarks of COVID-19 pathology, but also show for the first time that *not one but all* the major structural proteins, S, M, N and E, are able to activate the complement and kinin systems. Although therapeutics that target the complement (11, 12) and the kinin systems (9) have been promising, thorough understanding of the interplay between complement and coagulation systems in COVID-19 pathophysiology will still be requisite if we are to design therapeutic interventions to treat not only active patients but also the “long-haulers”. More importantly however, since SARS-CoV-2 undergoes frequent mutations to generate a new strain or a new variant, computational models and mathematical algorithms, that identify mutation-prone sites in the sequence of each of the structural proteins, may also help in the design of vaccines ahead of an impending SARS-CoV-2-variant pandemic.

## Data Availability Statement

The raw data supporting the conclusions of this article will be made available by the authors, without undue reservation.

## Author Contributions

AS, SM, and TW performed the complement experiments. WY, DR, and MF did the work on FXII and HK binding as well as the BK assay. BVG designed and HD and XX performed the SPR studies and BG designed and planned the experiments and helped with interpretation of the data. AK consulted on the kinin studies, and EP contributed with her expertise in blood coagulation and helped with the discussion and interpretation of data as well as writing the manuscript. All authors contributed to the article and approved the submitted version.

## Funding

This work was supported in part by grants from the National Institutes of Allergy and Infectious Diseases R01 AI 060866, R01 AI-084178, R56-AI 1223476 (to BG), from the National Institute of General Medical Sciences R01 GM121511 and Terry C. Johnson Cancer Center (to BVG), and the NIH/NCI cancer support grant P30 CA008748 (to MSKCC).

## Conflict of Interest

BG and EP receive royalties from the sale of anti-gC1qR mAbs and gC1qR detection assay kit.

The remaining authors declare that the research was conducted in the absence of any commercial or financial relationships that could be constructed as a potential conflict of interest.

## Publisher’s Note

All claims expressed in this article are solely those of the authors and do not necessarily represent those of their affiliated organizations, or those of the publisher, the editors and the reviewers. Any product that may be evaluated in this article, or claim that may be made by its manufacturer, is not guaranteed or endorsed by the publisher.
